# Sex differences in age‐to‐maturation relate to sexual selection and adult sex ratios in birds

**DOI:** 10.1002/evl3.156

**Published:** 2020-01-13

**Authors:** Sergio Ancona, András Liker, M. Cristina Carmona‐Isunza, Tamás Székely

**Affiliations:** ^1^ Departamento de Ecología Evolutiva, Instituto de Ecología Universidad Nacional Autónoma de México Ciudad de México 04510 México; ^2^ MTA‐PE Evolutionary Ecology Research Group University of Pannonia PO Box 158 Veszprém 8201 Hungary; ^3^ Department of Limnology University of Pannonia PO Box 158 Veszprém 8201 Hungary; ^4^ Milner Centre for Evolution, Department of Biology & Biochemistry University of Bath Bath BA2 7AY UK; ^5^ Department of Evolutionary Zoology and Human Biology University of Debrecen H‐4010 Debrecen Egyetem tér 1 Hungary

**Keywords:** Adult sex ratio, age at maturation, life histories, phylogenetic path analyses, sexual dimorphism, sexual selection

## Abstract

Maturation (the age when organisms are physiologically capable of breeding) is one of the major life history traits that have pervasive implications for reproductive strategies, fitness, and population growth. Sex differences in maturation are common in nature, although the causes of such differences are not understood. Fisher and Lack proposed that delayed maturation in males is expected when males are under intense sexual selection, but their proposition has never been tested across a wide range of taxa. By using phylogenetic comparative analyses and the most comprehensive dataset to date, including 201 species from 59 avian families, we show that intense sexual selection on males (as indicated by polygamous mating and male‐skewed sexual size dimorphism) correlates with delayed maturation. We also show that the adult sex ratio (ASR), an indicator of the social environment, is associated with sex‐specific maturation because in species with a female‐skewed ASR, males experience later maturation. Phylogenetic path analyses suggest that adult sex ratio drives interspecific changes in the intensity of sexual selection which, in turn, influences maturation. These results are robust to alternative phylogenetic hypotheses and to potential life‐history confounds, and they provide the first comprehensive support of Fisher's and Lack's propositions. Importantly, our work suggests that both social environment and mate competition influence the evolution of a major life history trait, maturation.

**Impact Summary**

Maturation times have major fitness consequences by influencing longevity and the number of breeding opportunities, and males and females often mature at different ages. This is attributed to selection favoring divergent maturation optima among sexes, but the selective factors driving maturation bias are controversial and have remained elusive. Here, we report the most comprehensive analyses of maturation yet carried out, using data from 201 wild bird populations. We document that intense sexual competition associates with delayed maturation in the sex subjected to this selection. We also show that males mature later than females in female‐skewed populations, whereas male‐skewed environments associate with females maturing later than males. Notably, adult sex ratio, a proxy of social environment, drives sexual competition, which in turn influences maturation. Our findings have fundamental implications for both sexual selection and life‐history theory because they posit that strong sexual competition and surplus of the opposite sex promote the evolution of delayed maturation.

Age at sexual maturation is one of the most important life‐history traits of organisms because it greatly affects fitness by influencing the number of reproductive opportunities and survival (Roff 2002) and, consequently, mating success in males and fecundity in females (Stearns and Koella 1986). Therefore, individuals are expected to mature at the point along their developmental trajectories where their fitness is maximized (Stearns and Koella 1986). Sex differences in maturation are common in nature, and can be striking. For example, in numerous birds and mammals, including humans, males mature several months (or years) later than females, whereas in insects, fishes, and amphibians, it is common for females to mature considerably later than males (Fairbairn 2013). Sex differences in maturation are attributed to selective factors operating differently on males and females, which may lead to divergent optima for the sexes (Fairbairn 2007), and may tend to facilitate sexual conflict (Boulton et al. 2016). Nevertheless, the nature of these selective forces and thus the ultimate causes of maturation bias, are controversial and not fully understood (Wiley 1974; Stamps and Krishnan 1997).

Sexual selection may lead to sex difference in maturation age (Vollrath and Parker 1992) because the greater the intensity of competition for access to mates, the more it may pay individuals to continue growing and delay maturation until they are better prepared to compete for mates with fully mature individuals (Wiley 1974). Although this idea was put forward in seminal works by Fisher (1930) and Lack (1968), and is often used to explain delayed maturation in males relative to females in polygynous animals (Orians 1969; Post et al. 1999), it has not been tested across a wide range of taxa for over 40 years. Here, we test this proposition using birds as model organisms in phylogenetically controlled statistical analyses.

First, we test whether maturation bias relates to intensity of sexual selection using two indices of sexual selection: frequency of polygamy and extent of sexual size dimorphism (SSD). Polygamy and SSD are both thought to be indicators of strong sexual selection. On the one hand, polygynous mating is usually associated with strong mating skew and large variation in mating success in males (Shuster 2009), and thus imposes strong selection on traits that influence male mating success (Møller and Pomiankowski 1993; Shuster 2009). Similarly, polyandry could impose strong sexual selection on females, for example, in sex‐role reversed species (Clutton‐Brock 2007; Kvarnemo and Simmons 2013). On the other hand, the extent of SSD is used as an indicator of sexual selection since larger body size often confers advantages to males in mate competition and mate choice (Andersson 1994), and conversely, female‐skewed SSD may arise in species where females compete strongly for mates (Clutton‐Brock 2007; Székely et al. 2007). Therefore, we expect the more polygamous sex to attain sexual maturity later than the less polygamous sex, and the larger sex to mature later than the smaller sex.

In addition, we test whether breeding opportunities, as indicated by the adult sex ratio (ASR, usually expressed as proportion of males in the adult population; Ancona et al. 2017), influence maturation. Recent studies suggest that the ASR is related to survival, breeding system, and sex roles in birds and humans (Székely et al. 2014a; Liker et al. 2013; Schacht et al. 2017); hence, it is reasonable to assume that ASR may also influence maturation, although we are not aware of any study that has tested this hypothesis. As male‐skewed social environments may indicate female scarcity and intense male–male competition (Le Galliard et al. 2005) in male‐skewed populations, males are expected to postpone maturation to compete successfully for mates at an older age (Rodd et al. 1997). Conversely, a female‐skewed ASR is expected to promote the evolution of delayed maturation in females.

To test associations between maturation bias, sexual selection, and social environment, we use a comprehensive dataset that includes 201 bird species from 59 families and analyze this dataset by Phylogenetic Generalized Least Squares (PGLS; Freckleton et al. 2002). In addition, using phylogenetic path models (Gonzalez‐Voyer and von Hardenberg 2014) we compare the fit of five hypothesized scenarios to the data that represent plausible direct and indirect relationships between maturation bias, sexual selection, and social environment.

## Methods

### DATA COLLECTION

We conducted an extensive literature search to assemble published data on age of sexual maturation, body size, and sexual competition for males and females, as well as data on ASR currently available for wild bird populations (see Supporting Information External Database S1).

We systematically looked for data on age of sexual maturation for males and females in reference works (e.g., The Birds of the Western Palaearctic 2006 and Birds of North America 2015) and the primary literature through the Web of Knowledge and Google Scholar, using scientific and English names of specific taxa in combination with “age at maturity,” “age at first reproduction,” and “recruiting age.” First, we focused our search on 187 species for which Székely et al. (2014a) assembled data on male and female body size, sexual competition, and ASR in order to maximize the completeness of the dataset with regard to the working hypotheses. Data on male and female maturation are uncommon, and the number of species showing sex differences in maturation was limited in the initial dataset. Therefore, we extended the sampling to all other bird species that may exhibit sex differences in maturation according to AnAge, a curated online database of vertebrate life histories (De Magalhães et al. 2009). We accessed all original sources referenced in AnAge but used only those data on sexual maturation that came from peer reviewed handbooks and scientific publications. We obtained data on age of sexual maturation (in months) for a total of 201 species. The age of sexual maturation was often defined as age of first breeding, although other criteria were also used, including the level of gonadal maturity and the age when secondary sex characteristics are fully developed. We aimed at using the best data available, and we are not aware of any systematic bias that would undermine our working hypotheses. When several estimates were available for a given species (e.g., from different subspecies or from different studies), we used the ones based on largest sample sizes. We express maturation bias as log_10_(male age of maturation/female age of maturation).

We augmented the data on body size and sexual competition for both sexes originally assembled by Székely et al. (2014a). Body size was the mean body mass in grams of adult males and adult females, and SSD was computed as log_10_(adult male mass/adult female mass). Our dataset includes SSD estimates for the 199 species whose male and female maturation ages are known. We scored the frequency of polygamy for each sex on a 5‐point scale (0–4) where ``0'' denotes no incidence of polygamy or very rare polygamy (<0.1% of individuals), ``1'' rare polygamy (0.1–1%), ``2'' uncommon polygamy (1–5%), ``3'' moderate polygamy (5–20%), and ``4'' common polygamy (>20%; see details in Liker and Székely 2005; Liker et al. 2014). When frequency of polygamy was not provided in the original source, we scored the frequency of polygamy based on verbal descriptions of the mating behavior and pair bonds available for focal species. Since continuous data on frequency of polygamy was rare, scoring was essential to include as many species as possible. Scoring was carried out independently by two observers, and their scoring was highly consistent (intraclass correlation between two observers, *r*_ICC_ = 0.914, *F* = 22.2, *P* < 0.001, *n* = 28 species). When different indices of polygamy were available for a focal species, we used their average value. Following previous studies, we estimated polygamy bias as the difference between male and female polygamy scores (Liker and Székely 2005; Liker et al. 2013; Liker et al. 2014). Polygamy bias was estimated for all 201 species for which we obtained both male and female maturation ages.

The dataset originally assembled by Székely et al. (2014a) includes 187 species and uses the information on ASR currently available for birds. We obtained data on male and female maturation for 176 species listed in this initial dataset. In addition, we looked for ASR data for those 14 species for which information on both male and female maturation was available. Our final dataset includes 183 species for which we have both ASR and maturation data. We followed the criteria of Székely et al. (2014a) for extracting ASR data from literature. When several ASR estimates were available for a species (e.g., from different years or different populations), we used their average value. We used ASR estimates obtained by different methods, including censuses of individually marked breeding adults, captures of breeding and non‐breeding birds, counts of birds dying from natural causes (e.g., storms), counts of museum specimens, and demographic analyses (such as Veran and Beissinger 2009; Kosztolányi et al. 2011). ASR estimates obtained from different populations and gathered by different methods tend to provide consistent results (Székely et al. 2014a; Liker and Székely 2005; Liker et al. 2014), but to be on the safe side, we excluded ASR estimates based on counts of hunted birds, and preferred estimates that were least influenced by anthropogenic impacts such as habitat loss. We used ASR estimates as provided by the original sources, although for 15 species we computed ASR from tables or figures in the original data source that reported the number of adult males and females in a given population. Consistent with previous studies, ASR was estimated as the number of adult males/(number of adult males + number of adult females) (Ancona et al. 2017) and it was arcsine‐square‐root‐transformed before analysis (Székely et al. 2014a; Liker et al. 2013).

### STATISTICAL ANALYSES

#### Phylogenetic *generalized least squares*

We evaluated each correlation of interest, and its robustness to the addition of potential confounding effects in four steps. In step 1, we tested whether maturation bias is correlated with polygamy bias, SSD, or ASR using separate bivariate PGLS with maximum likelihood estimates of Pagel's λ values (Pagel 1997; Freckleton et al. 2002). To represent the phylogenetic relationships between species, we used the most comprehensive avian phylogeny that includes all 201 species in our dataset. To test the sensitivity of results to phylogenetic uncertainty, we used a sample of 1000 phylogenetic tress, which we extracted randomly from the 10,000 alternative phylogenetic hypotheses available at http://birdtree.org, using the sample tool offered on this website. One thousand trees are suggested as a robust sample to reduce potential errors associated with phylogenetic uncertainty (Rubolini et al. 2015). All phylogenetic trees were fully resolved (i.e., did not have polytomy) and included branch lengths (see details in Jetz et al. 2012). We repeated each PGLS model with each of the 1000 trees and calculated the mean ± standard errors (SE) of these 1000 repeats for the slope and the two‐tailed significance levels of the phylogenetic regressions. Then we calculated the distribution of slopes and *P*‐values for all bivariate phylogenetic regressions; we report these values in Supporting Information Fig. S1.

In step 2, we tested the robustness of the aforementioned bivariate correlations to potential confounds, following the rationale we developed in recent phylogenetic studies (Liker et al. 2014; Vági et al. 2019). To achieve this, we performed three multipredictor PGLS regressions to test whether polygamy bias, SSD, and ASR remain significant correlates of maturation bias after controlling for the potential confounding effects of three life history traits: chick developmental mode, adult mortality bias, and adult body mass. We included these three potentially confounding variables in multipredictor PGLS because enhanced somatic growth and lower rates of mortality often correlate with delayed maturation (Roff 2002), and relatively large‐brained precocial species tend to reach sexual maturation later than their altricial counterparts (Scheiber et al. 2017). Thus, each multipredictor model included these three life history traits along with one of the three predictors of interest, that is, polygamy bias, SSD, or ASR. Data on offspring developmental mode were collected from the published literature, categorized as (0) altricial, (1) semi‐altricial or semi‐precocial, or (2) precocial, and included as a three‐level factor in PGLS. Mortality data come from field studies in which annual mortality rates of adult males and adult females were estimated in the same population and using the same method (capture–recapture, ringing recoveries, or local return rates). Adult mortality bias was estimated as log_10_(adult male mortality/adult female mortality). Body mass (in grams) was computed as the mean masses of male and female adults and log_10_‐transformed before the analyses.

In step 3, we tested whether the intensity of sexual selection, as indicated by either polygamy bias or SSD, and ASR have additive effects on maturation bias. To achieve this, we fitted two sets of multipredictor PGLS. The first set of PGLS contained both polygamy bias and ASR as predictors of maturation bias. The second set of PGLS included SSD and ASR as predictors of maturation bias. Finally, in step 4, we investigated whether additive effects of the intensity of sexual selection and ASR on maturation bias remain significant after accounting for potential confounding effects of life history traits. To accomplish this, we repeated these two sets of multipredictor PGLS and included chick developmental mode, adult mortality bias, and body mass as additional predictors of maturation bias. We did not include polygamy bias and SSD simultaneously in multipredictor PGLS because collinearity is expected between these two indices of sexual selection, which may prevent a meaningful analysis of their effects in a single model.

We confirmed that the assumptions of PGLS analyses were met in the fitted models (Mundry 2014); see details in Supporting Information. To produce comparable effect sizes, we calculated standard PGLS parameter estimates (slopes) by scaling the predictors according to the method proposed by Gelman (2008). Different data availability for different variables prevented us from using the same sample sizes in all PGLS. All reported significance values are two‐tailed because we performed PGLS that did not assume directionality in the relationships between variables. We calculated mean slopes and significance levels of main terms included in multipredictor PGLS. We calculated the variance inflation factor (VIF) for each model based on standard linear models, because as far as we know VIF cannot be computed directly from PGLS models; VIF was <1.5 for all models, suggesting that multi‐collinearity may not inflate results. PGLS were carried out in R (R Core Team 2013, version 2.15.2) using the packages ``ape'' (Paradis et al. 2004, version 3.0‐8) and ``caper'' (Orme et al. 2012, version 0.5).

#### Phylogenetic path analyses

**Figure 1:**
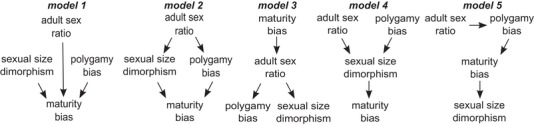
Phylogenetic path analyses. Schematic illustration of five hypothesized scenarios between maturation bias, polygamy bias, sexual size dimorphism, and the adult sex ratio.

In addition to PGLS, we also carried out phylogenetic confirmatory path analyses (Gonzalez‐Voyer and von Hardenberg 2014) to estimate statistical support and magnitude of hypothesized causal connections between maturation bias, polygamy bias, SSD, and ASR, using the R package ``phylopath'' version 1.0.1 (van der Bijl 2018). Phylogenetic confirmatory path analysis is a robust statistical tool to assess the goodness of fit of hypothesized direct and indirect associations among variables and thus to disentangle likely causal links between variables in comparative studies (Gonzalez‐Voyer and von Hardenberg 2014). First, we tested a multiple regression model in which maturation bias is directly influenced by all predictors, that is, polygamy bias, SSD, and ASR (Model 1). We also tested the alternative hypothesis that ASR is not directly influencing maturation bias, but instead is the causal agent of SSD (Lovich et al. 2014) and polygamy bias (Liker et al. 2013; Liker et al. 2014) and these two factors have direct effects on maturation bias (Model 2). Additionally, since maturation bias can also influence ASR because the late‐maturing sex takes longer to join the adult population and thus can be underrepresented in the adult population (Székely et al. 2014b; Lovich et al. 2014), we tested an alternative model hypothesizing that maturation bias may have a direct influence on ASR, which in turn remains as the causal agent of SSD and polygamy bias (Model 3). We tested a fourth alternative model hypothesizing that both ASR and polygamy bias directly influence SSD, and this latter factor in turn leads to maturation bias (Model 4). Finally, we tested a fifth model considering a hypothetical direct effect of ASR on polygamy bias, which in turn directly influences maturation bias, and making maturation bias the causal parent of SSD (Model 5). See Figure 1 for a schematic illustration of the five hypothesized scenarios for the direct and indirect associations between maturation bias, polygamy bias, sexual size dimorphism, and the adult sex ratio we tested.

Phylogenetic confirmatory path analyses were run with three phylogenies randomly selected from the 1000 trees used in PGLS to test the sensitivity of results to phylogenetic uncertainty. A path model was considered to have an acceptable fit to the data when the Fisher's *C* statistic was not statistically significant (>0.05; Gonzalez‐Voyer and von Hardenberg 2014). We determined the best fitting path model from the model set using the *C* statistic Information Criterion adapted to path analysis and adjusted for small sample sizes (CIC_c_); the best fit was given by the model with the lowest CIC_c_ score (Gonzalez‐Voyer and von Hardenberg 2014). Differences between any particular path model *i* and the best‐fitting model larger than 2 units and 10 units in their CIC_c_ scores (Δi > 2 and >10) are considered to be acceptable and very strong support, respectively, for a better fit by the best model to the data (Gonzalez‐Voyer and von Hardenberg 2014).

The above implementation of phylogenetic path analysis (Gonzalez‐Voyer and von Hardenberg 2014; van der Bijl 2018) accounts for phylogenetic nonindependence by constructing a series of bivariate linear PGLS models in which one variable is treated as the dependent variable, and the other is the independent variable. However, in PGLS the estimate of phylogenetic signal λ depends on the defined direction of the relationship. In path analysis, however, correlations between pairs of variables are the input so the directionality of the statistical model should not be an issue. To overcome this problem, we repeated the analyses using the approach proposed by Santos (2012) that does not rely on directional models to calculate nondirectional correlations. In brief, we (1) determined the phylogenetic signal (λ) separately for each variable by maximum likelihood method as implemented in the ``pgls'' function of R package ``caper'' (note that these latter models did not contain predictor variables), (2) used this variable‐specific λ value to re‐scale the phylogenetic tree to a unit tree (using an R code developed by R.P. Freckleton), and (3) used the transformed tree to calculate phylogenetically independent contrasts for the variable by the ``pic'' function of the ``ape'' R package. We repeated this process for each variable (using the variable‐specific λ value) then used these phylogenetically transformed values of the variables for fitting the path models (Santos 2012). We used the R package ``piecewiseSEM'' (Lefcheck 2016) to fit the same path models to the data as described above, and evaluated model fit by *C* statistics for each fitted model, where a statistically nonsignificant result means acceptable fit. We compared the fit of the path models by their CICc values.

## Results

### SOCIAL POLYGAMY, SEXUAL SIZE DIMORPHISM, AND MATURATION

Fifty out of 201 species (24.9%) exhibit maturation bias, and the difference between male and female maturation varies widely. For example, males mature 48 months earlier than females in the kakapo *Strigops habroptila*, but males mature 60 months later than females in the Australian bustard *Ardeotis australis* (see Supporting Information External Database S1).

**Figure 2:**
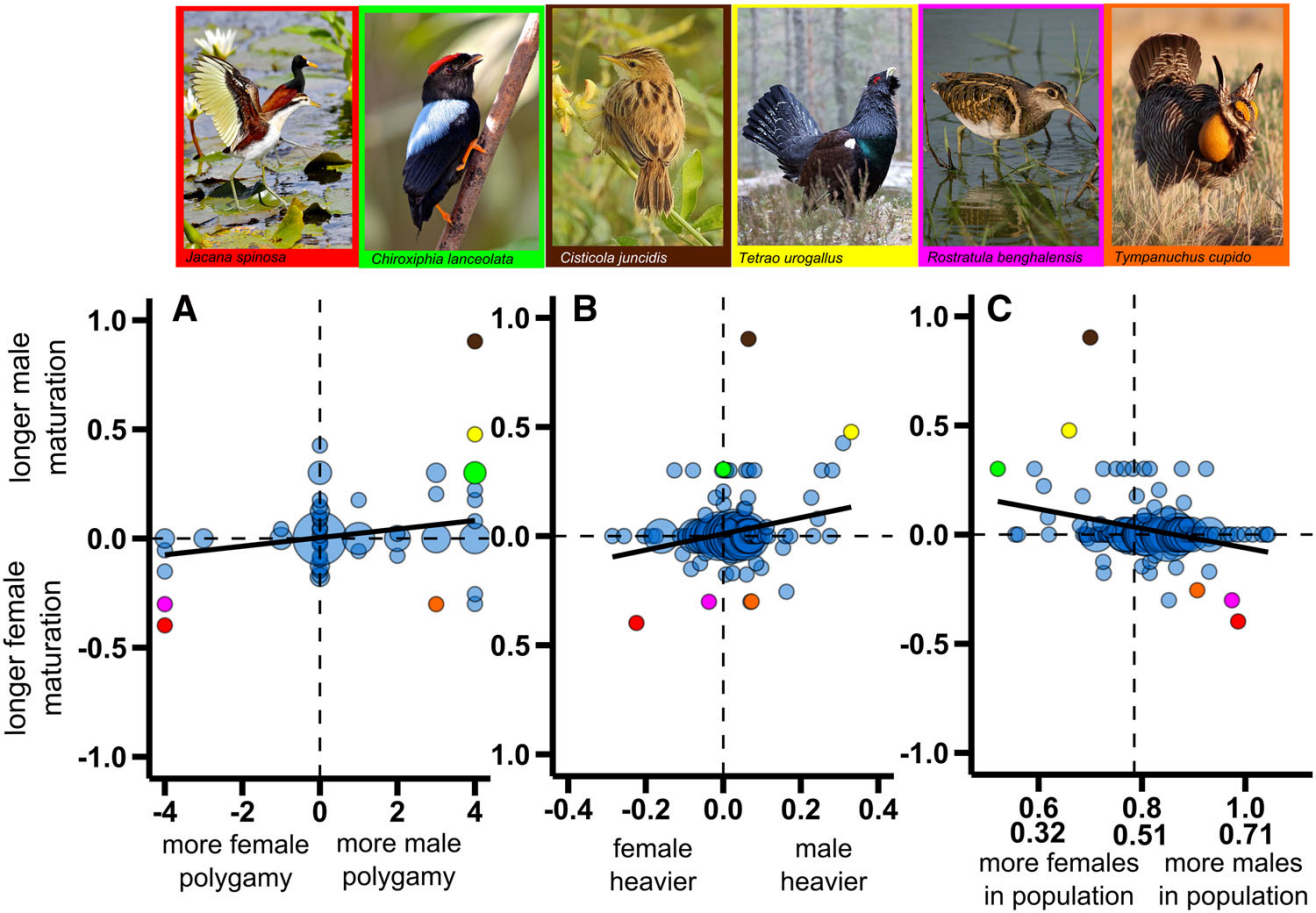
(A) Maturation bias is associated with polygamy bias in birds (mean [±SE] of 1000 phylogenetic generalized least squares (PGLS) models using different phylogenies: slope = 0.069 [<0.001], *P* < 0.001 [<0.001]; *n* = 201 species). Maturation bias was estimated as log(male age at maturation/female age at maturation). Polygamy was scored for each sex separately on a 5‐point scale, from 0 to 4, and polygamy bias was computed as male minus female polygamy score (see Methods section). Point size is proportional to the sample size of each data point showing 1–75 observations. (B) Maturation bias is associated with sexual size dimorphism in birds (slope = 0.129 [<0.001], *P* < 0.001 [<0.001]; *n* = 199 species). Sexual size dimorphism was estimated as log(adult male mass/adult female mass). Point size is proportional to the sample size of each data point showing one to five observations. (C) Maturation bias in relation to the adult sex ratio (slope = −0.081 [<0.001], *P* < 0.001 [<0.001]; *n* = 183 species). Adult sex ratio (ASR) was estimated as the proportion of males in the adult population (arcsine‐square‐root‐transformed). The regression (solid) lines show mean slope fitted by phylogenetic regressions using 1000 different phylogenies. In panel (C), second *x*‐axis labels correspond to the back transformed ASR (even ASR shown by vertical dotted line). Point size is proportional to the sample size of each data point showing one to five observations. Different colored points on all plots show where the exemplified species in photos appear in each plot. For further details of these relationships, see also Supporting Information Figure S1. Photo credits from left to right: *Jacana spinosa* © G. Friesen; *Chiroxiphia lanceolata* by © G. Friesen; *Cisticola juncidis* by Afsarnayakkan (https://bit.ly/2HnllT0), used under CC BY‐SA 4.0, cropped and rescaled from original; *Tetrao urogallus* by sighmanb (https://bit.ly/2Hnl1DM), used under CC BY 2.0, cropped and rescaled from original; *Rostratula benghalensis* by J. Thompson (https://bit.ly/2HjGg9x), used under CC BY 2.0, cropped and rescaled from original; *Tympanuchus cupido* by © S. Henkanaththegedara.

Polygamy bias is associated with maturation bias, because the more polygamous sex attains sexual maturity at an older age than the less polygamous sex (Fig. 2A). Importantly, polygamy bias remains a significant predictor of maturation bias when we include potentially confounding life history variables in the model such as offspring developmental mode, adult mortality bias, and body mass (Supporting Information Table S1A). Consistent with these results, maturation bias is also correlated with sexual size dimorphism, since the larger sex matures later than the smaller sex (Fig. 2B). Importantly, the phylogenetically controlled association between maturation bias and SSD remains robust to potential effects of life history variables (Supporting Information Table S1B).

### ADULT SEX RATIO AND MATURATION

Social environment is also associated with maturation bias since female‐skewed ASRs are associated with delayed maturation in males relative to females, and male‐skewed ASRs are associated with delayed maturation in females relative to males (Fig. 2C). The latter relationship between maturation bias and ASR remains significant when we control for the potential effects of chick developmental mode, adult mortality bias, and body mass (Supporting Information Table S1C).

### SEXUAL SELECTION, ASR, AND MATURATION

The intensity of sexual selection, as indicated by either polygamy bias or SSD, and the ASR has additive effects on maturation bias. First, polygamy bias and ASR remain significant predictors of maturation bias in multipredictor models (mean [±SE] of 1000 PGLS: polygamy bias: slope = 0.056 [<0.001], *P* = 0.005 [<0.001]; ASR: slope = −0.054 [<0.001], *P* = 0.005 [<0.001]; *n* = 183 species). Importantly both polygamy bias and ASR remain significant predictors when potential life‐history confounds are included in the model (Supporting Information Table S2A). Second, SSD and ASR remain significant correlates of maturation bias in multipredictor analyses (SSD: slope = 0.097 [<0.001], *P* = 0.002 [<0.001]; ASR: slope = –0.041 [<0.001], *P* = 0.032 [<0.001]; *n* = 181 species). Notably, ASR remains a significant predictor of maturation bias, although SSD becomes marginally nonsignificant when we account for the effects of life history confounds in multipredictor analyses (Supporting Information Table S2B).

### PHYLOGENETIC PATH ANALYSES

**Figure 3:**
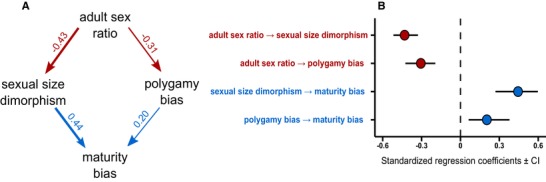
Path diagram (A) and standardized regression coefficients ± 95% confidence intervals (B) of the phylogenetic path model with strongest support by our data (Model 2). In panel (A), red and blue arrows indicate negative and positive relationships, respectively, and numbers represent standardized regression coefficients. In panel (B), red and blue dots indicate negative and positive relationships, respectively. Three randomly selected phylogenies provided similar results.

A single path model fits the data best (Table 1), and this result is consistent between two modeling approaches (see Supporting information Table S3). According to the best‐fitting model, female‐skewed ASRs direct the evolution of male‐skewed SSD and male polygamy, which in turn lead to delayed maturation in males relative to females. Conversely, male‐skewed ASRs favor the evolution of female‐skewed SSD and female polygamy, and these conditions promote evolution of delayed maturation in females relative to males (Fig. 3A and B).

**Table 1:** Maturation bias in relation to polygamy bias, sexual size dimorphism, and adult sex ratio using Phylogenetic Path Analyses. See Figure 1 for the structure of path models. The analyses were run with three different phylogenies randomly selected from the 1000 trees used in PGLS. Models are listed according to CIC_c_ values, from lowest to highest. Only model 2 (marked in bold) had strong support in our data, and the three different phylogenies provided consistent results. *C*: Fisher's *C* statistics, *k*: number of independence claims, *q*: number of parameters, ΔCIC_c_: difference in CIC_c_ scores from the best fitting model, *w*: CIC_c_ weights

Model	*C*	*k*	*q*	*P*‐value	CIC_c_	ΔCIC_c_	*w*
**2**	**5.981**	**2**	**8**	**0.201**	**22.818**	**0.000**	**1.0**
4	36.804	3	7	<0.001	51.451	28.633	0.0
3	48.009	3	7	<0.001	62.656	39.838	0.0
5	62.612	3	7	<0.001	77.259	54.441	0.0
1	118.498	3	7	<0.001	134.849	112.031	0.0

## Discussion

Our comparative analyses support Fisher's (1930) and Lack's (1968) postulations that intense sexual competition in males selects for delayed male maturation, since males attain sexual maturity later when they are more polygamous, and thus experience more intense sexual selection (Shuster 2009), than females. And our results also suggest that females may delay maturation when they are more polygamous, and thus experience more intense sexual selection than males. Maturation bias may emerge as a result of sexual selection acting on developmental trajectories, with prolonged maturation allowing individuals to fully develop the physiological, morphological, and behavioral capabilities required for fighting, courting, and breeding successfully (Wiley 1974).

Maturation bias is also associated with the extent of sexual size dimorphism in birds, since the larger sex matures later than the smaller sex, as it does in insects, fishes, turtles, and mammals (Alexander et al. 1979; Thornhill and Alcock 1983; Gibbons and Lovich 1990; Bisazza 1993). The relationship between maturation bias and SSD in birds provides additional support for the proposition that sex differences in age‐to‐maturation evolve when one sex experiences more intense sexual selection than the other sex (Lack 1968; Orians 1969). On the one hand, delayed male maturation relates to male‐skewed SSD, which often reflects strong sexual competition among males (Andersson 1994). On the other hand, delayed female maturation relates to female‐skewed SSD, which may evolve in species where females compete strongly for mates (Clutton‐Brock 2007; Székely et al. 2007).

We also show that in female‐skewed populations, males mature later than females, and in male‐skewed populations, it is females that mature later. Although opposed to our prediction, this result is also consistent with sexual selection theory. A skewed ASR may serve as an indicator of multiple mating opportunities for the rare sex (McNamara et al. 2000), and is associated with reduced care and increased polygamy in the rare sex in both birds and humans (Liker et al. 2013; Liker et al. 2014; Schacht et al. 2014). Thus, when females are in abundance, males can benefit from delaying maturation if it allows them to devote more time and energy to developing the traits that increase their success in mate competition. Conversely, when males are in abundance, females are expected to postpone maturation to enhance their competitive success at older ages (Székely et al. 2014b). This result is conceptually important because it provides the first comparative evidence that sexual maturation is influenced by the mating opportunities available to males and females in a given population.

Intense sexual competition promotes earlier male maturation (protandry) in spiders and insects (Vollrath and Parker 1992; del Castillo and Nuñez‐Farfán 1999), enabling males to arrive earlier at the best territories and encounter more unmated females (Morbey and Ydenberg 2001). However, in birds, we find support for the opposite argument that intense sexual competition among males favors the evolution of delayed male maturation (Fisher 1930; Lack 1968). Life history differences among invertebrate and vertebrate species may result in sexual selection favoring opposite maturation strategies. In invertebrates, adult lifespan is often short and generations rarely overlap, implying that newly matured males do not have to compete with older males. Instead, newly mature males compete with males of their own generation, and may succeed in mate competition by hastening maturation and being ready early for mating, especially if females mate only once (Thornhill and Alcock 1983; Vollrath and Parker 1992). In contrast, the life cycle of birds as of many vertebrates involves complex interactions between overlapping generations, and newly matured males often have to compete with older males with superior physical and/or behavioral characteristics. In this scenario, strong sexual competition is expected to favor delayed male maturation because early‐maturing males are likely to be outcompeted (Fisher 1930; Wiley 1974). We cannot discount, however, the alternative (or additional) possibility that ASR bias imposes high levels of mating competition on the supernumerary sex (Le Galliard et al. 2005), which responds by accelerating maturation. This process would be analogous to the way menarche is accelerated in female humans in response to limited mating opportunities and low availability of marital partners as indicated by female‐skewed ASRs (Ellis 2004). This could imply, for instance, that in female‐skewed environments females speed up maturation whereas males postpone maturation to be successful competitors at an older age.

The relationship between maturation bias and SSD could represent a causality dilemma because of the positive feedback that maturation bias may impose on SSD and vice versa. On the one hand, maturation bias may emerge as a result of SSD because the larger sex requires more time to grow, and therefore matures later than the smaller sex (Stamps and Krishnan 1997); whereas on the other hand, sexual selection may act directly on maturation (Gibbons et al. 1981; Stearns and Koella 1986) leading in turn to sex differences in adult body size (Stamps and Krishnan 1997). Size and age at maturity are closely related life history traits that may be direct targets of selection (Stearns and Koella 1986), so positive feedbacks between them could occur on an evolutionary time scale. Nonetheless, our confirmatory path analyses provide strong support to the hypothesis that SSD, and thus strong sexual selection, directs maturation bias rather than the reverse (Lack 1968).

Similarly, maturation bias may impose a positive feedback on polygamy bias and vice versa. According to sexual selection theory, maturation bias evolves as a response to sexual competition among males after the species has become polygamous (Lack 1968). Alternatively, maturation bias may evolve by natural selection when the optimal age‐dependent investment in reproduction differs between the sexes due to sex‐specific ecological and life history constraints, and delayed maturation promotes polygamy in the underrepresented and later‐maturing sex (Wiley 1974). Our confirmatory path analyses strongly support the former scenario, in which polygamy bias drives maturation bias (Lack 1968; Orians 1969).

An alternative explanation for the correlation between ASR and maturation bias is that the late‐maturing sex is underrepresented because it takes longer to join the adult population (Székely et al. 2014b; Lovich et al. 2014). This seems less likely because delayed maturation is associated with lower relative adult mortality in our data (Supporting Information Table S1C), which should increase the proportion of the late‐maturing sex in the adult population (Székely et al. 2014a). Moreover, results of path analyses strongly suggest that ASR bias is a driver, rather than a consequence of maturation bias in birds. Nevertheless, maturation bias may impose an evolutionary feedback on ASR and vice versa, and this feedback would not be detected by the path analyses. Experiments are probably needed to further probe whether ASR influences maturation rather than the reverse and better envisage the underlying mechanisms underpinning this association.

Importantly, age of sexual maturation is often defined as age of first breeding in avian studies (and not by the actual physiological states of individuals), and thus delayed maturation may be partially due to low availability of breeding spots or mates in focal populations (Wiley 1974). Further comparative and empirical studies focused on the proximate and ultimate causes of sex differences in maturation and life histories would benefit enormously from accurate estimations of physiological states of individuals.

In conclusion, we have demonstrated that in birds, the ASR, an indicator of perceived mating opportunities (Székely et al. 2014b; Liker et al. 2013), directs the intensity of sexual selection, which in turn favors the evolution of delayed maturation by sex. These results are consistent with comparative studies in birds showing that under female‐skewed ASR, males are larger and more polygamous than females, whereas under male‐skewed ASR, females are larger and more polygamous than males (Székely et al. 2014a; Liker et al. 2014). Comparative analyses of taxa that exhibit marked variation in maturation times, sex ratios, body size, and sexual selection (e.g., spiders, fishes, mammals) are needed to understand whether the patterns we report from birds may hold across invertebrates and vertebrates.

Associate Editor: A. Charmantier

## Supporting information


**Fig. S1**. Related to Figs. 2A, 2B and 2C.
**Table S1**. Maturation bias (response variable) in relation to polygamy bias, sexual size dimorphism or adult sex ratio when life‐history confounds (chick developmental mode, adult mortality bias and adult body mass) are accounted for in Phylogenetic Generalised Least Squares models (PGLS).
**Table S2**. Analyses testing the robustness of significant additive effects of sexual selection (as indicated by either polygamy bias or SSD) and ASR on maturation bias to the effects of life‐history confounds.
**Table S3**. Supplementary results of phylogenetic path analyses using the method proposed by Santos (2016).

Supporting Information
