# Supplementary material for: Sex differences in age‐to‐maturation relate to sexual selection and adult sex ratios in birds
Source: Evol Lett. 2020 Jan 13;4(1):44–53. doi: 10.1002/evl3.156 (PMC7006465; doi:10.1002/evl3.156)
Supplement: Supplementary file 1 — Fig. S1. Related to Figs. 2A, 2B and 2C. Table S1. Maturation bias (response variable) in relation to polygamy bias, sexual size dimorphism or adult sex ratio when life‐history confounds (chick developmental mode, adult mortality bias and adult body mass) are accounted for in Phylogenetic Generalised Least Squares models (PGLS). Table S2. Analyses testing the robustness of significant additive effects of sexual selection (as indicated by either polygamy bias or SSD) and ASR on maturation bias to the effects of life‐history confounds. Table S3. Supplementary results of phylogenetic path analyses using the method proposed by Santos (2016). [file EVL3-4-44-s002.docx]

**Supplementary Materials for**

**Sex differences in age-to-maturation relate to sexual selection and adult sex ratios in birds**

Sergio Ancona, András Liker, M. Cristina Carmona-Isunza and Tamás Székely

**This section includes:**

Checks of assumptions of PGLS

Figure S1

Tables S1-S4

**Additional Supplementary Materials:**

External Database S1 (Separate Excel file)

**Checks of assumptions of PGLS**

Preliminary inspection of correlations did not reveal any non-linear tendency in the relationships between maturation bias and its proposed predictors. Scaling as well as, the log and arcsine-square-root-transformations of the continuous variables prevented the occurrence of skewed distributions or outliers. The original mean ± standard deviation values of variables that were scaled or transformed (see details in Methods) are as follows: maturation bias: 1.104 ± 0.586; polygamy bias: 0.781 ± 1.756; sexual size dimorphism: 1.076 ± 0.247; adult sex ratio: 0.538 ± 0.085; adult mortality bias: 0.972 ± 0.362; species’ mean body mass: 1004.75 ± 2956.91. The number of cases was at least 11 times higher than the number of predictors in all PGLS; the minimum acceptable ratio for the #cases/#of predictors is 10 (Mundry 2014). The number of cases for different levels of the categorical predictors ranged from 9 to 113, thus rare levels were not an issue. Inspection of fitted regressions using standard linear models revealed nor skewedness for residual distributions, neither occurrence of influential data points.

**Fig. S1**. Related to Figs. 2A, 2B and 2C. Distribution of slopes (red histograms) and p values (green histograms) of bivariate PGLS models fitted with 1000 different phylogenetic trees. A – B: maturation bias vs. polygamy bias; C – D: maturation bias vs. sexual size dimorphism (SSD); E – F: maturation bias vs. adult sex ratio (ASR).

**Table S1.** Maturation bias (response variable) in relation to polygamy bias, sexual size dimorphism or adult sex ratio when life-history confounds (chick developmental mode, adult mortality bias and adult body mass) are accounted for in Phylogenetic Generalised Least Squares models (PGLS). Slope and p values are shown as the mean [± SE] of 1000 multi-predictor PGLS with different phylogenies. Detailed description of variables and analyses are provided in Methods.

| **Models and predictor variables** | **slope [± SE]** | **p [± SE]** | **No. of species** |
| --- | --- | --- | --- |
| (A) *Polygamy bias* *and life-history confounds* | | | |
| **Polygamy bias** | **0.061 [<0.001]** | **<0.001 [<0.001]** | 139 |
| Developmental mode | -0.019 [<0.001] | 0.388 [<0.001] |  |
| Adult mortality bias | -0.029[<0.001] | 0.087 [<0.001] |  |
| Body mass | 0.029 [<0.001] | 0.187 [<0.001] |  |
| (B) *Sexual size dimorphism and* *life-history confounds* | |  |  |
| **Sexual size dimorphism** | **0.048 [<0.001]** | **0.009 [<0.001]** | 139 |
| Developmental mode | -0.005 [<0.001] | 0.714 [0.002] |  |
| Adult mortality bias | -0.016 [<0.001] | 0.368 [0.002] |  |
| Body mass | 0.002 [<0.001] | 0.942 [0.003] |  |
| (C) *Adult sex ratio* *and* *life-history confounds* | |  |  |
| **Adult sex ratio** | **-0.072** **[<0.001]** | **<0.001 [<0.001]** | 134 |
| Developmental mode | 0.001 [<0.001] | 0.980 [<0.001] |  |
| **Adult mortality bias** | **-0.044 [<0.001]** | **0.015 [<0.001]** |  |
| Body mass | -0.005 [<0.001] | 0.807 [<0.001] |  |

**Table S2.** Analyses testing the robustness of significant additive effects of sexual selection (as indicated by either polygamy bias or SSD) and ASR on maturation bias to the effects of life-history confounds. We report slope and p values as the mean [± SE] of 1000 multi-predictor PGLS with different phylogenies. Detailed description of variables and analyses are provided in Methods.

| **Models and predictor variables** | **slope [± SE]** | **p [± SE]** | **No. of species** |
| --- | --- | --- | --- |
| (A) *Polygamy bias*, *adult sex ratio* *and* *life-history confounds* | | | |
| **Polygamy bias** | **0.045 [<0.001]** | **0.016 [<0.001]** | 134 |
| **Adult sex ratio** | **-0.055 [<0.001]** | **0.005 [<0.001]** |  |
| Developmental mode | **-**0.006 [<0.001] | 0.783 [<0.001] |  |
| **Adult mortality bias** | **-0.048 [<0.001]** | **0.007 [<0.001]** |  |
| Body mass | 0.007 [<0.001] | 0.747 [<0.001] |  |
| (B) *Sexual size dimorphism, adult sex ratio* *and* *life-history confounds* | | | |
| Sexual size dimorphism | 0.036 [<0.001] | 0.070 [<0.001] | 134 |
| **Adult sex ratio** | **-0.060 [<0.001]** | **0.002 [<0.001]** |  |
| Developmental mode | 0.016 [<0.001] | 0.673 [0.010] |  |
| **Adult mortality bias** | **-0.043 [<0.001]** | **0.020 [<0.001]** |  |
| Body mass | -0.007 [<0.001] | 0.690 [0.005] |  |
|  |  |  |  |

| Model | *C* | *df* | *P*_c_ | CIC_c_ | ΔCIC_c_ |
| --- | --- | --- | --- | --- | --- |
|  |  |  |  |  |  |
| **2** | **7.944** | **4** | **0.094** | **29.763** | **0.000** |
| 3 | 20.380 | 4 | 0.000 | 35.979 | 6.216 |
| 4 | 23.481 | 6 | 0.001 | 43.921 | 14.158 |
| 5 | 51.527 | 6 | 0.000 | 73.617 | 43.854 |
| 1* | - | 0 | - | - | - |
|  |  |  |  |  |  |

**Table S3.** Supplementary results of phylogenetic path analyses using the method proposed by Santos (2016). See Fig. 1 for the structure of path models. Models are listed according to CIC_c_ values, from lowest (most supported) to highest. Only model 2 (marked in bold) had support in our data, similarly to the analyses presented in Table 3. *C*: Fisher’s *C* statistics, *df*: model’s degrees of freedom, *P_c_*: significance of *C* statistic (P > 0.05 indicates acceptable model fit), ΔCICc: difference in CICc scores from the best fitting model. *Saturated model with 0 *df*, hence model fit is not evaluated in piecewiseSEM because tests of directed separation are not possible.
